# Prevalence and influencing factors of anxiety, depression, and burnout among teachers in China: a cross-sectional study

**DOI:** 10.3389/fpsyt.2025.1567553

**Published:** 2025-03-20

**Authors:** Lulu He, Lingling Huang, Yuanyuan Huang, Hehua Li, Ziyun Zhang, Junhao Li, Shisong Lin, Kai Wu, Dongchang Huang, Fengchun Wu

**Affiliations:** ^1^ The Affiliated Brain Hospital, Guangzhou Medical University, Guangzhou, China; ^2^ Dongguan Dalang No. 1 Middle School, Dongguan, China; ^3^ School of Biomedical Sciences and Engineering, South China University of Technology, Guangzhou, China; ^4^ Dalang Town Experimental Primary School, Dongguan, China; ^5^ Guangdong Engineering Technology Research Center for Translational Medicine of Mental Disorders, Guangzhou, China; ^6^ Key Laboratory of Neurogenetics and Channelopathies of Guangdong Province and the Ministry of Education of China, Guangzhou Medical University, Guangzhou, China

**Keywords:** school teachers, anxiety, depression, burnout, prevalence

## Abstract

**Background:**

Teachers are more likely to experience mental health issues than the general population, yet few studies focus on Chinese teachers. After the “Double Reduction” policy, teacher workload and stress have risen significantly. In Dongguan, a city actively implementing educational reforms, teacher mental health may be particularly concerning. Anxiety, depression, and burnout are prevalent mental health issues. This study examined the prevalence and factors linked to anxiety, depression, and burnout among Dongguan teachers.

**Methods:**

A cross-sectional survey evaluated the mental health of primary and secondary school teachers in Dongguan, China, using two-stage random sampling. Eleven schools were randomly selected, with 30 teachers chosen from each of 330 invited participants, 313 completed the survey (94.8% response rate), and 259 valid responses were retained after excluding incomplete or inconsistent answers. The study used the Zung Self-Rating Anxiety Scale (SAS), Zung Self-Rating Depression Scale (SDS), and Maslach Burnout Inventory-Educators Survey (MBI-ES). Wilcoxon rank-sum and Kruskal–Wallis tests identified factors linked to anxiety, depression, and burnout. Variables with *p* ≤ 0.10 in univariate analysis were included in multivariate logistic regression, with cut-offs of 50 for SAS, 53 for SDS, 27 for Emotional Exhaustion (EE), 13 for Depersonalization (DP), and 31 for Personal Accomplishment (PA). A *p* ≤ 0.10 threshold was used to avoid omitting potential predictors.

**Results:**

The study found anxiety, depression, and burnout prevalence rates of 19.3%, 34.7%, and 74.5%, respectively (95% CI: 14.5–24.1; 28.9–40.6; 69.2–79.9). Junior high school teachers (OR = 0.47) and those with higher education (OR = 0.39) had a lower depression risk. Female teachers (OR = 1.96) had a higher risk of emotional exhaustion (EE), while older teachers (OR = 0.923) had a lower risk of reduced personal accomplishment (PA). Anxiety and depersonalization (DP) showed no significant associations.

**Conclusion:**

The study found high rates of depression and burnout among teachers, influenced by educational level, school type, sex, and age. Authorities should address these issues by clarifying teacher responsibilities, providing mental health training, and establishing monitoring systems. As a cross-sectional study, it cannot determine causality, and further longitudinal research is needed to confirm these findings.

## Introduction

Teachers are at higher risk of developing common mental disorders than those in other occupations (1). A global systematic review indicated that, when only considering clinically meaningful psychological conditions, the prevalence of teachers’ burnout, anxiety, and depression was 25.12–74%, 38–41.2%, and 4–77% (2), respectively. Owing to differences in environmental (educational systems),cultural conditions between countries and differences in measurement instruments and sample characters, there were significant differences in the reported prevalence of these conditions among teachers (3). In China, the prevalence rates of depressive symptoms and anxiety among primary and secondary school teachers in the Sichuan, Jiangxi, and Shandong provinces were 20.41% and 26.44%, respectively (4), while anxiety prevalence among teachers in Henan Province was 13.67% (5), and burnout prevalence among university teachers reached 75.4% (6). Although the reported prevalence data vary, they consistently exceed the national norms for Chinese adults(12-month prevalence of depressive disorder:3.6%; anxiety disorder: 5.0%) (7), highlighting a concerning issue in teachers’ mental health in China. Teachers’ psychological well-being not only affects their personal lives but also has profound implications for students-the future generation. For instance, teachers’ burnout predicts teaching quality (8), and depressive symptoms in teachers are linked to reduced gains in students’ math skills (9). Additionally, teacher distress was found to be positively associated with internalizing symptoms and externalizing symptoms among elementary students (10).

Teachers may experience more severe mental health issues compared to individuals in other professions (11). The psychological problems of school teachers are caused by objective reasons, such as work pressure, salary problems, limited career development, etc. Beyond their teaching responsibilities, teachers are burdened with numerous non-teaching tasks, such as assessments, inspections, evaluations, and administrative duties, which are often repetitive and unrelated to their professional expertise (12). Furthermore, the implementation of “Double Reduction” policy (aimed at reducing students’ homework and after-school tutoring burdens), has introduced both new expectations and challenges for teachers. While the policy seeks to improve education quality, it has unintentionally increased teachers’ workloads, extended working hours, and heightened psychological stress, thereby exacerbating their mental health challenges (13).

As an economic powerhouse in the Pearl River Delta, Dongguan’s rapid economic growth has spurred significant educational demands. Teachers in Dongguan may face immense pressure and high expectations due to substantial investments in education. Moreover, the city’s large migrant population and frequent mobility might complicate the allocation of educational resources, leading to uneven distribution and increased work pressure for some teachers (14). Furthermore, as a frontrunner in actively implementing the “Double Reduction Policy”, Dongguan’s pioneering role in this educational reform requires teachers to adapt continuously (15). On November 17, 2020, Dongguan Municipality issued the “Special Work Notice on Reducing the Burden on Primary and Secondary School Teachers”, aimed at alleviating the workload of teachers in local schools. However, Liang’s research revealed that administrative documents related to non-teaching tasks (e.g., paperwork, inspections, and non-academic responsibilities) did not decrease—and even exhibited a slight upward trend—before and after the policy’s implementation (16). In summary, the mental health status of teachers in Dongguan may be concerning, but there is currently a lack of research on their psychological health status.

Anxiety, depression, and burnout are three common mental health issues experienced by teachers. To our knowledge, this study marks the first investigation into the prevalence rates and potential influencing factors of these conditions among primary and secondary school teachers in Dongguan, thereby addressing a gap in the existing literature. Given Dongguan’s implementation of policies aimed at reducing teacher workload, the prevalence rates reported in this study provide a critical reference point for future assessments of the psychological impact of these policies. However, longitudinal studies with multiple time points are essential to monitor policy-driven changes in teachers’ mental well-being. Concurrently, our findings establish comparative benchmarks for evaluating teacher mental health in cities undergoing similar socio educational transitions. Furthermore, the analysis of influencing factors can assist policymakers, schools, and society in identifying teacher subgroups at higher risk of mental health problems and developing targeted interventions to address their needs. Finally, this study seeks to answer the following questions: What is the prevalence of anxiety, depressive symptoms, and occupational burnout among teachers in Dongguan? What are the potential factors influencing the mental health of teachers in this region?

## Materials and methods

### Study design and participants

Between September and November 2023, an online questionnaire was distributed via Questionnaire Star through school administrations, who assisted in disseminating the survey link to primary and secondary school teachers in Dongguan, China, via WeChat groups, to conduct a cross-sectional survey on their mental health. The online questionnaire designed for data collection encompassed the demographic characteristics of the participants, work-related conditions, and three self-rated psychological scales. The purpose and application of the study were elucidated at the beginning of the online questionnaire. All participants provided informed consent online before accessing the questionnaire by completing a digital consent form that outlined the study’s purpose, procedures, and confidentiality measures. Only after agreeing to the terms of the consent form were participants able to proceed to the questionnaire. Each participant was permitted to respond to the questionnaire only once. The questionnaire was completed voluntarily, and personal information was kept confidential. This study was approved by the Ethics Committee of the Affiliated Brain Hospital, Guangzhou Medical University.

### Eligibility criteria

Inclusion criteria: All full-time primary and secondary school teachers who have been serving for more than 6 months and who are currently in service were included in this study.

Exclusion criteria: Part-time teachers, teachers who are on maternity and sick leave for different reasons, and teachers who are acutely ill during the data collection period were excluded from this study.

### Calculation of sample size

The required number of samples for the present study was calculated using the formula, $\text{n }=\;\frac{{\text{Z}}_{\text{α}/2}\times \text{p}\times \left(1-\text{p}\right)}{{\text{d}}^{2}}$ , where n = the number of samples, ${\text{Z}}_{\text{α}/2}$ = 1.96, *p* = 0.26 (according to previous studies, the prevalence of anxiety, depression, and burnout among Chinese teachers was 20.41%, 26.44%, and 75.4%, respectively. When *p* = 0.26, n reaches its maximum value.) and d = 0.05 (margin of error). Based on this formula, the minimum sample size required for this study was determined to be 296 teachers.

### Sampling

A random sampling method was employed to select primary and secondary school teachers from the Dongguan area. The sampling process consisted of two stages. Initially, we selected 11 schools from 608 primary and secondary schools in Dongguan using a random number table. We contacted Headteachers at the selected schools and informed them about the study before conducting investigation. Subsequently, within each of the 11 selected schools, 30 teachers was recruited. Teachers were randomly selected using a random number table based on their employee identification numbers. If the Headteacher declines to cooperate or the school has fewer than 30 teachers, we will use a random number table to select the next school and document the reason for replacement. If a teacher fails to meet the inclusion criteria, we will select the next teacher using a random number table and record the reason for substitution. Participation was voluntary, and teachers who declined to participate were excluded from the study. 17 teachers chose not to participate in the study and were thus excluded. Consequently, the final sample was made up of 313 teachers, with a response rate of 94.8% (313 out of 330). These participants were recruited from different geographical areas within Dongguan.

### Questionnaire

The online questionnaire was structured into three main sections: demographic information (e.g., age, gender), work-related details (e.g., teaching experience, school type, subjects taught), and three self-rated psychological scales, including SAS, SDS, and MBI-ES. To ensure data quality, the questionnaire implemented mandatory response settings to prevent missing data. Anxiety symptoms were assessed using the SAS. Based on the Chinese normative data for teachers, the cutoff value of the SAS standard score was defined as follows: no anxiety (≤ 49 points), mild (50–59 points), moderate (60–69 points), and severe anxiety (≥ 70 points) (17–19). Depressive symptoms were assessed using the SDS. In accordance with the results of the Chinese norm, the cut-off value of the SDS standard score was defined as follows: no depression (≤ 52 points), mild (53–62 points), moderate (63–72 points), and severe depression(≥ 73 points) (17, 19). Zhang revised and validated the SAS and SDS scales for Chinese primary and secondary school teachers, confirming their reliability and validity as measures of anxiety and depression in this population (20). We employed the Chinese version of Maslach Burnout Inventory-Educator teachers Survey (MBI-ES) to appraise participants’ burnout syndromes. The MBI-ES comprises three subscales that measure EE DP, and PA. The MBI-ES includes 22 items: the EE subscale encompasses 9 items, the DP subscale consists of 5 items, and the PA subscale comprises 8 items. An EE score of 27 or higher was regarded as high, and a DP score of 13 or higher was considered high. A PA score of 0–31 was considered low (21). The MBI-ES has an extremely high consistency and psychometric validity (22). A study by Wu revised the MBI-ES for the Chinese cultural context, confirming its three-factor structure (EE, DP, and PA) and demonstrating excellent reliability (23). Burnout was defined as reporting a high level of burnout on one or more subscales. That is, if any one of the following conditions is met: EE score ≥ 27, or DP score ≥ 13, or PA score ≤ 31, it is considered burnout (24).

### Data quality control

To identify potentially invalid questionnaires, we analyzed the distribution of response times and used the 5th percentile as the cut-off point. This approach is consistent with established methods for detecting insufficient effort responding (25). Questionnaires completed in less than 300 seconds (the 5th percentile of response times) were deemed invalid. Questionnaires that were not completed meticulously were eliminated using similar or opposite questions. For instance, with regard to “I fall asleep easily and sleep well all night” in SAS and “I don’t sleep well at night” in SDS. After short completion times and contradictory answers, 54 questionnaires were deemed invalid. In total, 259 valid questionnaires were obtained.

### Statistical analysis

The data were imported into SPSS 27.0, for statistical analysis. The results of the demographic and work-related variables are described by frequency and proportion. The SAS, SDS, and MBI-ES scores of the participants are presented as bar charts. Since the majority of the SAS standard score, SDS standard score, and scores of the three burnout subscales were skewed data, the differences between groups were explored using the Wilcoxon rank-sum and Kruskal–Wallis tests. Finally, multivariate binary logistic regression was employed to explore the independent factors influencing anxiety, depression, and burnout. The continuous scores from the assessment instruments were dichotomized based on established cut-off points: a standard score of 50 or higher on the SAS indicated anxiety, a standard score of 53 or higher on the SDS indicated depression, and scores of 27 or higher for EE, 13 or higher for DP, and 31 or lower for PA on the MBI-ES indicated burnout. In the univariate analysis, many variables showed a trend toward significance(*p*< 0.10). These variables, along with those that were significant (*p* ≤ 0.05), were included as independent variables in the multivariate logistic regression equation. In this study, a significance level of *p* ≤ 0.10 was used for variable inclusion in the multivariate analysis. This decision was made to minimize the risk of Type II errors (false negatives) and to ensure that potentially relevant predictors were not excluded, particularly given the exploratory nature of our research. While a conventional threshold of *p*< 0.05 is commonly used, a more lenient threshold of *p* ≤ 0.10 has been employed in similar exploratory studies to capture a broader range of variables that may contribute to the model (e.g (26, 27).,). The variance inflation factor (VIF) was used to assess multicollinearity, and a cut-off value of five was established. When there was a multicollinearity problem, we selected one of the independent variables to enter into the regression equation. The assumption of linearity in the logit for the continuous variable (age) was assessed by the Box–Tidwell test (28). The –2 log-likelihood ratio test was used to test the overall significance of the model. The fit of the model was assessed by the Hosmer–Lemeshow goodness-of-fit Chi-square test. If there are missing values, we will employ listwise deletion for the analysis, followed by multiple imputations to assess the sensitivity of the results to missing values (29). Significance was defined as a *p* value ≤ 0.05.

## Results

### Sociodemographic characteristics of participants

We finally included 259 teachers. Over half the participants were women (79.2%), 73% were married, 88% had a bachelor’s degree, 75.7% were primary school teachers, and 78.8% were ordinary teachers without administrative positions. (**Table 1**).The demographic characteristics of our sample align with the broader population of teachers in Guangdong Province (Dongguan is part of Guangdong Province), as reported in the Educational Statistics Yearbook of China (2022). Specifically, women comprised 75.63% of primary school teachers, 59.90% of middle school teachers, and 56.91% of high school teachers, while bachelor’s degree holders accounted for 78.54%, 87.34%, and 81.58%, respectively. Our sample reflects these trends, with 79.2% women and 88% holding a bachelor’s degree, supporting its representativeness.

**Table 1:** Socio-demographics and work characteristics of the study population.

Variable	Category	Frequency	Percentage(%)
Sex	male	54	20.8
	female	205	79.2
Marital status	single	60	23.2
	married	189	73
	Divorced/separated	7	2.7
	other	3	1.2
Academic qualification	junior college	16	6.2
	Bachelor	228	88
	Master	15	5.8
School type	elementary	196	75.7
	Junior high	51	19.7
	Senior high	6	2.3
	Other	6	2.3
administrative position	School Administrator	8	3.1
	Middle level cadres	41	15.8
	Ordinary teachers	204	78.8
	Other	6	2.3
Knowledge Areas	social science	163	62.9
	natural science	68	26.3
	other	28	10.8
Age group	20-29 years	70	27
	30-39 years	80	30.9
	40-49 years	85	32.8
	50 years or more	24	9.3
Service year	Under 5 years	80	30.9
	6-9 years	38	14.7
	10-19 years	50	19.3
	20-29 years	70	27
	30 years or more	20	7.7

### Anxiety, depression, and burnout among teachers

**Figure 1:**
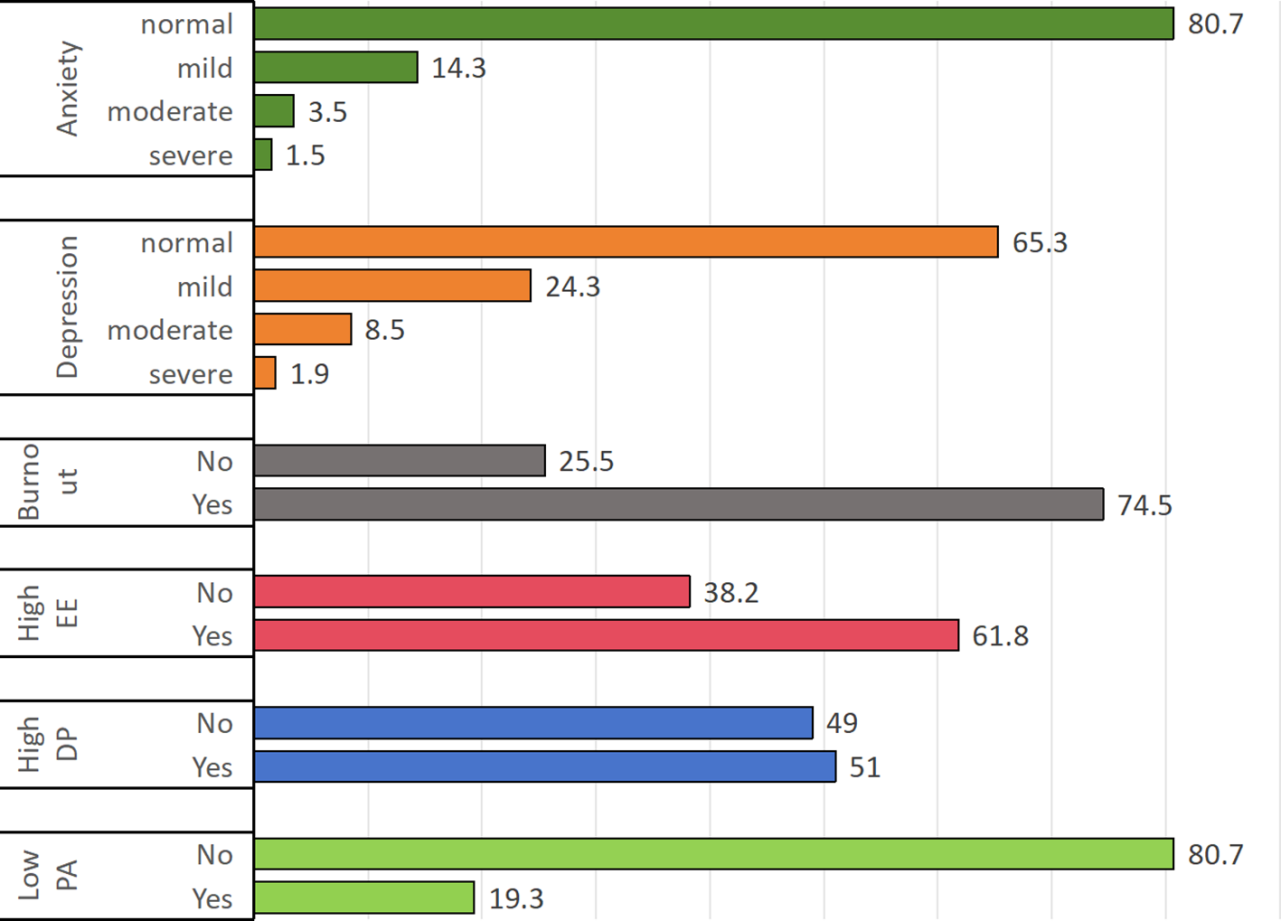
Prevalence of anxiety, depression and burnout.

The prevalence rates of anxiety and depressive symptoms were 19.3% and 34.7%, respectively. The prevalence of burnout syndrome was 74.5%, with 61.8% experiencing EE, 51% experiencing DP, and 19.3% experiencing reduced PA. The prevalence of moderate-to-severe anxiety and depression among teachers was 5% and 10.4%, respectively. (**Figure 1**).

### Factors influenced the level of anxiety, depression, and burnout among teachers

Univariate analysis revealed significant differences in anxiety symptoms among different school types(e.g., primary schools, middle schools, and high schools), depressive symptoms between different education levels(e.g., junior college’s degree, bachelor’s degree, and above) and school types, EE between different sexes and marital statuses(e.g., single, married, divorced), DP between different marital statuses, and reduced PA between different marital statuses and administrative positions(e.g., school Administrator, middle level cadres, general teachers). (**Table 2**)

**Table 2:** Univariate analysis to identify influencing factors associated with anxiety, depression and burnout syndrome.

Variable	Category	SAS standard score			SDS standard score			EE score			DP score			PA score		
		M(Q1,Q3)	Z/H	*p*	M(Q1,Q3)	Z/H	*p*	M(Q1,Q3)	Z/H	*p*	M(Q1,Q3)	Z/H	*p*	M(Q1,Q3)	Z/H	*p*
Age	20-29	40 (31,48)	3.101	0.376	50 (36,59)	2.945	0.4	34 (27,41)	14.892	**0.002**	14 (10,16)	7.673	0.053	35 (29,41)	16.75	**0.001**
	30-39	37 (33,45)			45 (38,54)			28 (21,36)			13 (9,15)			38 (32,43)		
	40-49	39 (34,48)			48 (38,55)			29 (24,35)			11 (9,14)			39 (35,43)		
	≥50	39 (33,46)			45 (35,54)			26 (22,31)			11 (9,15)			40 (37,47)		
Service year	≤5	38 (31,46)	1.425	0.84	48(36,58)	1.479	0.83	32(23,38)	9.209	0.056	13(10,15)	7.539	0.11	37(30,42)	21.273	**<0.001**
	6-9	40(34,49)			48(40,58)			33(22,40)			13(9,16)			34(31,39)		
	10-19	38(33,46)			45(38,54)			26(23,33)			12(8,14)			39(37,46)		
	20-29	39(34,47)			49(38,56)			30(24,36)			11(9,14)			40(34,43)		
	≥30	41(33,50)			45(36,54)			27(23,33)			13(10,15)			40(37,48)		
Sex	male	36(33,45)	-1.331	0.183	43(38,53)	-1.275	0.202	26(21,34)	-2.292	**0.022**	13(9,15)	-0.29	0.772	38(33,42)	-0.366	0.714
	female	40(34,48)			49(38,58)			31(24,37)			13(9,15)			38(33,42)		
Marital status	single	42(33,48)	2.36	0.501	50(39,59)	3.662	0.3	33(26,40)	10.45	**0.015**	14(11,16)	8.51	**0.037**	35(30,41)	10.935	**0.012**
	married	38(33,46)			46(36,54)			28(23,35)			11(9,15)			39(33,43)		
	Divorced/ separated	44(31,53)			49(35,59)			26(24,42)			9(9,13)			48(32,49)		
	other	34(34,/)			41(40,/)			37(27,/)			10(9,/)			34(32,/)		
Academic qualification	junior college	40(34,44)	5.186	0.075	50(41,59)	9.376	**0.009**	26(20,34)	5.12	0.077	10(8,15)	4.869	0.088	40(35,42)	0.574	0.751
	Bachelor	39(33,48)			48(38,56)			30(24,37)			13(9,15)			38(33,42)		
	Master	33(26,41)			39(30,44)			23(20,32)			10(8,14)			33(31,42)		
School type	elementary	40(34,48)	10.938	**0.012**	49(38,58)	7.919	**0.048**	30(24,37)	5.661	0.129	11 (9,15)	5.267	0.153	38 (33,43)	4.933	0.177
	Junior high	36 (30,41)			40 (35,51)			28 (21,35)			14 (10,15)			39 (32,41)		
	Senior high	44 (40,51)			54 (50,58)			37 (27,39)			16 (10,17)			36 (31,38)		
	Other	34 (30,37)			45 (41,47)			25 (20,29)			12 (6,14)			34 (28,38)		
Knowledge Areas	social science	39 (33,48)	1.64	0.441	46 (36,56)	0.228	0.892	30 (24,37)	2.138	0.343	13 (9,15)	0.651	0.722	38 (33,42)	2.048	0.359
	natural science	40 (34,45)			49 (37,56)			30 (23,38)			12 (9,15)			38 (32,43)		
	other	37 (31,45)			47 (41,55)			27 (22,36)			13 (10,14)			37 (30,42)		
Administrative position	School Administrator	36 (32,45)	2.164	0.539	38 (32,50)	6.792	0.079	28 (21,37)	0.58	0.901	11 (6,15)	2.229	0.526	43 (39,46)	9.402	**0.024**
	Middle level cadres	36 (32,45)			43 (35,52)			29 (24,35)			11 (9,14)			39 (34,42)		
	general teachers	40 (33,48)			49 (38,56)			30 (23,38)			13 (9,15)			38 (32,42)		
	other	40 (32,63)			51 (46,65)			29 (25,41)			13 (9,16)			35 (26,38)		

M, median; Q1, Lower Quartile; Q3, Upper Quartile.

Bold values indicate statistical significance at *p*< 0.05

One-way analysis of variance using the Wilcoxon rank-sum test or Kruskal–Wallis test was employed to assess differences in psychometric test scores among categories.

### Multivariate logistic regression

Variables with *p* ≤ 0.10 in univariate analysis were included as independent variables in multivariate logistic regression analysis. For the logistic regression models, continuous scores from the SAS, SDS, EE, DP, and PA scales were dichotomized based on established cut-off values: SAS ≥ 50, SDS ≥ 53, EE ≥ 27, DP ≥ 13, and PA ≤ 31. To assess multicollinearity, variance inflation factor (VIF) values were calculated for all independent variables. In the models with SAS, SDS, and DP as dependent variables, the VIF values ranged between 1.00–1.01, 1.01–1.36, and 1.04–1.66, respectively, indicating no significant multicollinearity. However, in the models with EE and PA as dependent variables, multicollinearity was observed between age and years of teaching experience (VIF = 7.34 and 6.97 for age, and VIF = 6.35 and 6.46 for years of work, respectively). To address this, only age was included in the multivariate regression equation, as it was deemed more clinically relevant (2, 30, 31). The VIF values for other independent variables in these models ranged between 1.01–1.67, indicating no significant interactions between variables. Moreover, the age met the linearity assumption. There is no missing data due to the mandatory response settings.

Model with SDS standard score as the dependent variable: H-L test, *p* > 0.05; model chi-square = 26.07, *p*< 0.001. Model with EE score as the dependent variable: H-L test, *p* > 0.05; model chi-square = 14.98, *p* = 0.02. Model with PA score as the dependent variable: H-L test, *p* > 0.05; chi-square = 23.43, *p* = 0.001. The results of the multivariate logistic regression showed that a high education level (OR = 0.39) and being a junior high school teacher (OR = 0.47) were protective factors for depression, being female (OR = 1.96) was a risk factor for EE, and older age (OR = 0.923) was a protective factor for reduced PA. (**Table 3**)

**Table 3:** Results of independent influencing factors of Depression, Emotional exhaustion and Personal accomplishment in the binary logistic regression model.

Variable	B	SE	Wald χ2	*p*	OR^a^	95% CI for OR	
						lower	upper
Depression							
Administrative position							
General teachers					Ref^b^		
Middle level cadres	-0.57	0.4	2.05	0.152	0.56	0.26	1.24
School Administrators	-0.67	0.83	0.64	0.424	0.51	0.1	2.63
School type							
elementary					Ref^b^		
Junior high	-0.76	0.38	4.03	**0.045**	0.47	0.22	0.98
Senior high	1.13	0.88	1.63	0.202	3.08	0.55	17.36
Academic qualification	-0.94	0.44	4.56	**0.033**	0.39	0.16	0.93
Emotional exhaustion							
Sex							
Male					Ref^b^		
Female	0.67	0.34	4.03	**0.045**	1.96	1.02	3.79
Age	-0.01	0.02	0.06	0.805	1	0.96	1.03
Academic qualification	0	0.39	0	0.994	1	0.47	2.15
Marital status							
Single					Ref^b^		
Married	-0.64	0.4	2.61	0.106	0.53	0.24	1.15
Divorced/separated	-1.29	0.89	2.08	0.150	0.28	0.05	1.59
Personal accomplishment							
Age	-0.08	0.03	9.47	**0.002**	0.92	0.87	0.97
Marital status							
Single			0.18	0.98	Ref^b^		
Married	0.03	0.42	0.01	0.943	1.03	0.45	2.36
Divorced/separated	0.51	1.23	0.18	0.676	1.67	0.15	18.39
Administrative position							
General teachers			1.44	0.696	Ref^b^		
Middle level cadres	-0.19	0.54	0.13	0.719	0.82	0.29	2.37
School Administrators	-18.88	14039.86	0	0.999	0	0	/

a OR, odds ratio.

b ref, reference.

Bold values indicate statistical significance at *p*< 0.05.

## Discussion

To the best of our knowledge, this is the first cross-sectional survey of the prevalence and factors influencing primary and secondary school teachers’ anxiety, depression, and burnout in Dongguan, China. While similar studies have been conducted in other regions of China (e.g., Sichuan, Jiangxi, and Shandong), Dongguan serves as a distinctive research setting, characterized by rapid urbanization, a substantial transient population, and proactive implementation of both the “Double Reduction” policy and teacher burden reduction initiatives. These characteristics may influence teachers’ mental health and burnout levels in ways that differ from other regions. Our investigation into teacher mental health serves dual roles in addressing a critical gap in the literature (1): providing a critical reference point for evaluating the psychological impacts of educational reforms, and (2) offering comparative benchmarks for urban centers undergoing comparable demographic transitions characterized by rapid urbanization and high migrant flows. The current research results indicated that 19.3%, 34.7%, 74.5% of teachers exhibited symptoms of anxiety, depression, and burnout, respectively. The results of the multivariate regression suggested that a high education level (OR = 0.39, 95%CI 0.16-0.93, *p* = 0.033) and teaching in junior high schools (OR = 0.47, 95%CI 0.22-0.98, *p* = 0.045) were independent protective factors of depression among teachers and being female (OR = 1.96, 95%CI 1.02-3.79, *p* = 0.045) and younger (OR = 0.92, 95%CI 0.87-0.97, *p* = 0.002) were independent predictors of teachers’ burnout.

Our findings indicate that the prevalence rates of moderate-to-severe (clinically meaningful) anxiety, moderate-to-severe depressive symptoms, and overall burnout among teachers were 5%, 10.4%, and 74.5%, respectively. Compared with the prevalence of depressive disorder among Chinese adults (6.8%) (32), the prevalence of clinically meaningful depression among teachers may be much higher. The prevalence of moderate-to-severe depressive symptoms and overall burnout was consistent with the results reported in a systematic review conducted by 2022 (2). However, the prevalence of moderate-to-severe anxiety found in this study was much lower than the results of this systematic review (38%–41.2%). The reason for this large difference may be attributed to several factors. First, the scales used for measurement differed: the 24 studies on anxiety included in the review mostly used the Depression, Anxiety, and Stress Scale (DASS), while our study employed the SAS. Second, the choice of cut-off values may have played a role. Specifically, the relatively low prevalence of anxiety in our study may be partially due to our use of a higher cut-off score (a standard score of 50, equivalent to a raw score of 40), in contrast to the lower cut-off score of 36 (raw score), commonly used for clinical screening, which tends to have a higher false-positive rate (18). Additionally, regional cultural and policy factors may have contributed to this disparity, meaning that teachers in Dongguan may indeed have lower anxiety levels compared to the global average. The unique socio-economic and cultural context of Dongguan, along with specific burden-reduction policies implemented by the local government to alleviate teachers’ workload and improve their well-being, might contribute to the lower anxiety levels observed in this region. Therefore, to further validate these findings, future studies could employ multiple scales to measure anxiety and compare the results across different cultural and regional contexts.

In terms of prevalence rates, the data on the prevalence rates of the three dimensions within occupational burnout are also worth exploring,: EE (61.8%) > DP (51%) > reduced PA (19.3%). Burnout is characterized by emotional exhaustion, depersonalization, and reduced personal accomplishment. EE refers to the feelings of stress and exhaustion. DP implies that individuals try to prevent the consumption of emotional energy by regarding others as objects or numbers rather than people (15). Reduced PA involves a negative self-evaluation of work, including feeling incompetent and professionally incapable. In contrast to the reports of Nada and Inmaculada (33), who found that low PA was more common than high EE or DP among teachers, our results suggest a different pattern. According to Sziget’s two-factor model of teachers’ burnout, EE and DP scores primarily reflect general burnout, while PA scores more specifically indicate a sense of personal accomplishment (34). Teachers in Dongguan exhibited high levels of general burnout but maintained relatively stable PA scores. This may be attributed to the role of emotional labor, which helps teachers maintain close student-teacher relationships and classroom quality (35, 36), thereby preserving their sense of accomplishment. However, emotional labor can also exacerbate EE and DP over time, as the effort to manage emotions may lead to increased psychological strain (37). This dual role of emotional labor—protective in the short term but potentially harmful in the long term—can be understood through the lens of the Job Demands-Resources model (38). Within this framework, emotional labor functions both as a demand (increasing exhaustion) and a resource (preserving PA). The intensification of Emotional EE and DP can also lead to a decline in PA (39). Over time, when the negative impact of EE and DP on PA (emotional labor as a demand) outweighs the protective effect of emotional labor on PA (emotional labor as a resource), PA may decline significantly, as seen in other studies (40, 41). The differences between our findings and those of Nada and Inmaculada may reflect cultural or contextual variations in teachers’ roles and responsibilities, particularly regarding their emphasis on student-teacher relationships and classroom quality.

Our study found that high educational level is a protective factor against depression among teachers, which is consistent with the majority of previous research (42, 43). However, some other studies hold the opposite view (44). For example, a study related to depressive symptoms among Egyptian teachers showed that the higher the educational attainment, the higher the depression score; it was believed that high educational attainment affected depressive symptoms through occupational stress (45). These contradictory results may have been caused by different assessment scales and regional cultural environments. We believe that the negative correlation between depression and educational attainment is likely to hold in China. Research on the teacher population suggests that teachers with undergraduate degrees perceive higher work pressure than teachers with master’s degrees (46), and such pressure is closely related to depressive symptoms (47) emotional regulation ability is positively correlated with educational attainment, meaning that teachers with lower educational levels may have poorer emotional regulation skills (48, 49). Furthermore, studies on the general population also support our view that depression is negatively correlated with educational attainment (50, 51), and the potential reasons are outlined below (1):depressed students had lower learning abilities, lagged in academic performance, and dropped out of school earlier, resulting in lower educational levels (52–56) (2).depressed children were more likely to have behavioral problems such as substance abuse, and behavioral problems were stronger predictors of educational attainment than depression (57) (3).compared to depressive symptoms influencing educational attainment, the influence of low educational attainment on the degree of depression may be more prominent among Chinese teachers. Peer unfriendliness, parental pressure, and teachers’ criticism may act as moderating mediators (58). Teachers with low educational attainment may have had poor academic performance during their school days and were more likely to be treated as unfriendly by their peers, parents, and teachers (4).educational attainment and depression may have a direct genetic causal relationship (59).

Our study revealed that primary school teachers exhibit a higher risk of depression compared to junior high school teachers, aligning with the findings of most prior research (2, 45). The high risk of depressive symptoms among primary school teachers may be attributed to the fact that they have high work demands while having relatively few resources (various supports and favorable factors related to teachers’ work), consequently resulting in high pressure on them (60). In terms of work demands, they had greater responsibilities (full responsibility for children’s physical, emotional, social, and intellectual development), played diverse roles (such as surrogate parents, social workers, counselors, and first responders), and had longer working hours (devoted more time and energy to caring for younger students) (45, 61). As they play diverse roles, they face greater role ambiguity and conflict. High role ambiguity and high role conflict will cause teachers to feel confused, uncertain, and contradictory at work; unclear about their responsibilities and expectations; and difficulty in coordinating various role requirements, thereby increasing psychological pressure and the risk of depression (47). Furthermore, the following reasons related to resources may also lead to more psychological problems for primary school teachers (1): primary school teachers lack relevant training when they face multiple roles (45) (2); compared with junior high school teachers, primary school teachers need to face more negative parental behaviors (62); (3) due to the younger age of students, students may pay less attention to their studies, which is also a decrease in teachers’ psychological support resources (63). Therefore, the mental health of primary school teachers deserves greater attention. When work demands cannot be reduced, schools, parents, and other institutions should provide more support to primary school teachers.

Notably, sex is an independent predictor of burnout. According to our findings, compared to men, women were more likely to experience exhaustion, which is consistent with many previous research results (64, 65). One possible reason is that women play more roles. Not only did they have heavy teaching tasks, but they were also responsible for taking care of the family, which led to greater pressure on female teachers. Moreover, women face special periods, such as pregnancy, childbirth, and menopause, and have a greater psychological burden (66). Women may also be more susceptible to emotions when facing pressure and challenges, resulting in a higher degree of exhaustion (67). Furthermore, compared to DP and low PA, teachers’ perceptions of safety and support were more strongly associated with exhaustion (68). Women may be more sensitive to the perception of safety and support, which partly explains why the difference between women and men only appeared in the dimension of EE rather than in the dimensions of DP or PA. However, some studies have reported different conclusions, believing that sex has nothing to do with exhaustion (68–70), or that men are more likely to experience exhaustion (71). Differences in scales for assessing burnout and differences in geographical, cultural or organizational factors may explain the contradictions in these results.

Besides sex, our results showed that age was an independent predictor of teachers’ burnout. The older the participant, the higher the PA scores. Most studies suggest that burnout is related to age, especially in the dimension of reduced PA (30, 31, 72, 73). Older teachers would accumulate more personal achievements and have more work experience, enabling them to better face the requirements and challenges at work (43). In contrast, young teachers face diverse sources of pressure. They may need to balance family responsibilities, social life, and career development simultaneously, which may distract them from their energy at work and result in less work being completed, thus affecting their sense of achievement (74). However, some studies believe that burnout is not related to age (34, 75). The age difference in burnout may only be a product of survivorship bias; that is, those who experienced burnout early in their careers may have already withdrawn from their careers. Therefore, only teachers with low burnout levels may engage in teaching (31). Thus, to clarify the relationship between age and PA more clearly and scientifically, future studies should exclude the influence of survivorship bias.

When summarizing the relationships between the aforementioned risk or protective factors and mental health outcomes, a common pattern emerges: demographic factors (e.g., sex, age) often impact teachers’ mental health through work-related factors such as role conflict, work pressure, and emotional labor. While demographic variables are relatively fixed, work-related factors like work pressure and support resources can be improved, which should be the focus of educational management. As mentioned above, female teachers and primary school teachers experience heightened pressure due to their multiple roles, increasing their risk of emotional issues. To address this, relevant authorities should actively revise and improve the current Teachers’ Law to clarify the boundaries of teachers’ responsibilities in teaching, administrative duties, and rest periods, thereby alleviating role conflict (76). Teachers’ emotional labor helps maintain relationships with students and classroom dynamics, sustaining their work performance (which may be reflected in the lack of significant decline in PA scores on the MBI-ES scale). However, this may mask their general burnout. Schools should intervene and provide supportive resources before teachers exhibit significant declines in work performance, which necessitates the establishment of mental health monitoring mechanisms (12). Pressure plays a primary mediating role in psychological issues. In line with the Communist Party of China Central Committee and the State Council’s guideline to reduce teachers’ burdens, local governments should minimize non-educational tasks and implement flexible working hours. Beyond material support, mental health training should be strengthened to enhance emotional regulation, especially for teachers with lower educational attainment (13).

This study has several limitations. First, regarding the study design, the cross-sectional nature of the research prevents us from establishing strong causal relationships between the study variables and anxiety, depression, or burnout syndrome. Additionally, all participants were recruited from one region of China (Dongguan), which limits the generalizability of our findings to teachers in other parts of mainland China. Furthermore, the scales used in this study, though validated and widely implemented in psychological health research, are screening tools rather than diagnostic instruments, meaning the prevalence rates of anxiety and depression may be overestimated due to the use of screening scales. While the online survey format could introduce potential biases, such as self-selection bias and response bias, our use of random sampling likely reduces the possibility of self-selection bias to some extent. Therefore, future research should adopt longitudinal designs, expand the study population to broader geographic regions, and incorporate clinical diagnostic follow-ups to address these limitations. Second, our actual sample size was slightly smaller than the minimum sample size calculated based on simple random sampling formulas, which may decreased representativeness, reduce the statistical power and precision of our findings. Finally, although this study explored several work-related variables (e.g., school type, subject taught) that may influence teachers’ psychological health, it did not include the measurement of a critical mediator—work pressure. Future studies could investigate the mediating effects of work pressure to provide a more comprehensive understanding of the mechanisms underlying teachers’ mental health issues. Despite these limitations, this study represents an important step forward, as there is limited research on the prevalence and associated factors of psychological problems among teachers in China.

## Conclusion

In conclusion, our study enriches the database on the mental health status of teachers in China and provides valuable insights into the psychological well-being of teachers in cities like Dongguan, which are undergoing rapid urbanization, actively implementing the “Double Reduction” policy. Our findings highlight the severity of mental health issues such as depressive symptoms and burnout among teachers in Dongguan and call for increased attention from relevant authorities, particularly for teachers who are less educated, teach in primary schools, are young, or are female. Additionally, given that our study used screening scales, had a relatively small sample size, and employed a cross-sectional design, future research should adopt longitudinal designs, incorporate clinically diagnostic instruments, and include larger samples.

## Data Availability

The raw data supporting the conclusions of this article will be made available by the authors, without undue reservation.
